# Perceived COVID‐19 health and job risks faced by digital platform drivers and measures in place to protect them: A qualitative study

**DOI:** 10.1002/ajim.23409

**Published:** 2022-06-28

**Authors:** Ellen MacEachen, Samantha B. Meyer, Shannon Majowicz, Pamela Hopwood, Meghan Crouch, Joyceline Amoako, Yamin T. Jahangir, Steve Durant, Antonela Ilic

**Affiliations:** ^1^ School of Public Health Sciences University of Waterloo Waterloo Ontario Canada

**Keywords:** covid‐19 pandemic, digital platforms, gig economy health interventions, occupational health and safety, precarious employment

## Abstract

**Introduction:**

As they deliver food, packages, and people across cities, digital platform drivers (gig workers) are in a key position to become infected with COVID‐19 and transmit it to many others. The aim of this study is to identify perceived COVID‐19 exposure and job risks faced by workers and document the measures in place to protect their health, and how workers responded to these measures.

**Methods:**

In 2020–2021, in‐depth interviews were conducted in Ontario, Canada, with 33 digital platform drivers and managers across nine platforms that delivered food, packages, or people. Interviews focused on perceived COVID‐19 risks and mitigation strategies. Audio recordings were transcribed verbatim and uploaded to NVivo software for coding by varied dual pairs of researchers. A Stakeholder Advisory Committee played an instrumental role in the study.

**Results:**

As self‐employed workers were without the protection of employment and occupational health standards, platform workers absorbed most of the occupational risks related to COVID‐19. Despite safety measures (e.g., contactless delivery) and financial support for COVID‐19 illnesses introduced by platform companies, perceived COVID‐19 risks remained high because of platform‐related work pressures, including rating systems. We identify five key COVID‐19 related risks faced by the digital platform drivers.

**Conclusion:**

We situate platform drivers within the broad context of precarious employment and recommend organizational‐ and government‐level interventions to prevent digital platform worker COVID‐19 risks and to assist workers ill with COVID‐19. Measures to protect the health of platform workers would benefit public health aims by reducing transmission by drivers to families, customers, and consequently, the greater population.

## 1 INTRODUCTION

Digital platform drivers, such as those working for Uber Eats, Amazon Flex, and Lyft, were busier than ever during the COVID‐19 pandemic as the public attempted to avoid infection by ordering take‐away food, shopping online, and taking ride‐hails rather than public transportation.^1^ Given airborne transmission of the virus and the nature of their work, this placed platform drivers in a position to become exposed to other people, infected with COVID‐19, and to transmit it to others as they moved food, packages, and people from one location to another.^2^ Although digital platform drivers operate between where people live (e.g., homes, care facilities) and wider communities, occupational and public health guidelines to protect these drivers from COVID‐19 exposure and to mitigate their role in disease transmission have been limited. Given that people act based on their understandings,^3^ we examined how the drivers understood and responded to perceived COVID‐19 risks at work. The aim of our Canadian study was twofold: 1) to identify, from the perspective of platform drivers, the perceived COVID‐19 exposure and job risks faced, and 2) to document the measures in place to protect the health of these workers and how workers responded to these measures.

The global digital platform economy continues to grow quickly. Mastercard and Kaiser^4^ describe an annual 17% growth rate of digital platforms that allow freelancers to connect with individuals or businesses for short‐term services or asset‐sharing and an expected gross volume of $455B USD by 2023. Of this, 88% of revenue is based on transportation‐based services such as ride‐hail and food or package delivery. During the pandemic, e‐commerce (i.e., requiring parcel delivery) made record gains; for instance, more than doubling in Canada over 2019–2020^5^ and growing by 12% in Europe.^6^ It is estimated that over 28M people in the EU work through digital platforms.^7^ In Canada, the number of digital platform workers is unclear; however, an administrative database study of gig work (defined as unincorporated self‐employed workers whose future business activity is uncertain, minor, or occasional) determined that gig workers comprised 8.2% of all workers in 2016.^8^

Digital platform work is precarious, low‐wage, and insecure.^9^ Precarious work has grown in recent decades and is related to the transformation of employment conditions as a result of technological advances and the increased mobility of capital and workers.^10^ It is generally characterized as involving contract renewal uncertainty, income inadequacy or volatility, and a lack of worker rights and protections and is associated with ill health.^11^, ^12^, ^13^ While some precariously‐employed workers have managers and workplaces, an added complexity facing platform workers is that they tend to be classified as self‐employed.^14^ This is despite the platforms having considerable and “employer‐like” control over the activities of these workers, including standardization of services and pay rates.^15^ The self‐employment status of digital platform workers has been steadily contested in the law courts across international jurisdictions^16^ and is the subject of a proposed European standard that, if passed, would provide a list of control criteria to determine whether the platform is an “employer.”^7^

Studies have identified ways in which digital platform gigs pose risk to workers' physical and mental health. Bartel et al.^17^ found that ride‐hail drivers, via encouraging nudges from their platform, drove for many hours without exiting the vehicle and experienced related back, leg, and foot pain. Due to limited bathroom access, drivers limited liquid intake to the point of dehydration. Their high amounts of sedentary behavior were often accompanied by unhealthy eating and weight gain. The study also found that mental health was affected by constant surveillance and the need to maintain adequate stores on platform rating systems. Similarly, others have noted that, while platforms promise flexibility and autonomy, the work actually involves unpredictability, work intensification, and financial hardship.^18^, ^19^ Nielsen et al.^20^ found that safety systems for platform workers were weak or absent.

Platform workers can be categorized into two main groups: web‐based (services performed remotely via the Internet) and location‐based (work in a specific geographical area).^21^ In our study, we were concerned with platform workers' interactions with the public and so focused on location‐based workers; specifically, those who delivered people, food, or packages. Because digital platform workers are mostly classified as self‐employed, they are legally responsible for their own health and safety, as they are considered as controlling their work and working conditions. As such, it is up to them, rather than a workplace manager, to both identify workplace health risks and find ways to avoid them. Unlike regular employers, platform companies (which tend to refer to themselves as “tech companies”) usually do not pay into social security systems, such as workers' compensation, employment insurance, and pension plans. As well, platform companies have mostly not been bound by labor laws that, for instance, require them to pay minimum wage, overtime, and provide holiday pay for workers and offer parental leave.^22^ This lack of social security protection leaves platform workers vulnerable and downloads the risks of this study onto workers themselves. For instance, studies find that digital platform drivers often earn below the minimum wage, when costs such as gas, car maintenance, and waiting periods are factored in.^23^, ^24^

Studies of platform workers find that, although platforms emphasize the flexibility of workers to choose when they work, this characterization underplays the active role that digital platform companies play in standardizing services and monitoring workers.^15^ Working times are mostly dictated by consumer demand and platform incentive payments and work is managed by the platform company to a significant degree.^21^ In 2021, the European Commission^7^ proposed a directive on improving working conditions in platform work. The directive proposes a list of control criteria to determine whether the platform is an “employer” with the aim of ensuring that people working through digital labor platforms are granted the legal employment status that corresponds to their actual work arrangements. The directive also calls for more transparency in algorithmic management. While the directive is subject to lobbying and change before final implementation, these initial proposals provide international policy leadership.

Interestingly, during the 2020–2021 waves of the COVID‐19 pandemic, and despite platform companies' practices of classifying workers as independent contractors and making claims of being only a “technology company” or “digital marketplace,”^25^ some platform companies engaged in further employer‐like roles by protecting worker health and safety through providing protective supplies to workers, such as hand sanitizer and face masks and offering some income compensation for workers when they were ill with COVID‐19.^19^, ^26^ During the pandemic, governments in many countries changed their stance toward self‐employed workers.^27^ For the first time, they became eligible for emergency government income support benefits, such as the Coronavirus Aid, Relief, and Economic Security (CARE) Act in the USA^28^ and, in Canada, the Canadian Emergency Response Benefit.^29^ In Canada, in early 2022, legislation was passed in Ontario that established a so‐called minimum wage for platform drivers.^30^ Although the Ontario government appeared to be engaging with strategies that provide some worker protection to platform drivers, the new minimum wage has been criticized as it applies only to “active” times (i.e., passengers in the car, delivery underway) and so does not cover the whole work day, including waiting times.^31^ Ultimately, the continued status of workers as self‐employed means that they remain excluded from occupational health and safety standards and most employment standards.

COVID‐19 has added a new layer of risk to the work of platform workers. Essentially, digital platform drivers are frontline, low‐wage, and self‐employed workers who, to date have had minimal occupational health guidance and support in the context of COVID‐19.^32^, ^33^

## 2 METHODS

A qualitative approach was used to address our research question regarding the nature of occupational health risks faced by platform drivers during COVID‐19 as core categories of analytic foci regarding platform driver COVID‐19 risk are still relatively unknown and undeveloped. The goal of qualitative research is not to identify distributions and typical characteristics for statistical generalization but to gain insight into social processes and complexities of human interaction.^34^ In this case, we were particularly interested in risk perceptions and decision‐making rationales related to perceived COVID‐19 risk management. While a recent mixed‐method study shed light on health and safety risk perceptions of platform drivers,^35^ their responses were limited to preset categories of anticipated response fields. By using in‐depth interviews with open‐ended questions, we were able to capture a wide range of risk perceptions of digital platform workers and managers in relation to key contexts of work pressures and platform occupational health interventions.

Our Ontario, Canada, based study used situational analysis,^36^ which considers events and behaviors in organizational and policy contexts, to capture the contextualized work and health perceptions of digital platform drivers. For the purpose of our study, we focused on digital platforms that delivered food (restaurant meal delivery), packages (parcel delivery; also, pharmacy/grocery delivery by workers who also shopped for the items as a part of the service), and people (ride‐hails), as these were very active during the pandemic. We use the term “drivers” in relation to all platform workers in this study, although some platform workers delivering food used a bicycle.

With our purposive sampling strategy,^37^ to be eligible, all participants needed to have worked during the COVID‐19 pandemic, and worker participants needed to have been engaged in digital platform work for at least 10 h per week for at least 1 month. Interviews with drivers were conducted by telephone or videoconference and focused on perceived risks related to COVID‐19 and mitigation strategies. A demographic survey was administered in interviews with worker participants. Interviews with platform managers obtained their insight regarding the organizational mitigation strategies. Relevant publicly available and participant‐supplied documentary data were also gathered. Altogether, the worker, manager and documentary data provide multiple‐source triangulation of information about the working conditions of drivers.^38^

Detailed field notes were made for each interview. Verbatim transcripts were verified and then coded by the research team using NVivo software. Coding was conducted by varied pairs of the research team and focused on both deductive codes (based on the interview guide) and inductive codes (arising from the data). The codes were then analyzed thematically to focus on key issues pertaining to digital platform work and COVID‐19‐related risks and mitigation strategies. The situational analysis focused on understanding the participant account in context and encompassed relevant policies at the organizational and governmental levels.

A nine‐person Stakeholder Advisory Committee composed of worker advocates, legal experts, and public health and government officials played an instrumental role in data recruitment schemes (reviewing interview questions, recruitment strategies), guiding our interpretation of the data and in formulating recommendations presented herein. Ethics approval for our study was provided by the University of Waterloo. Informed consent was obtained from all participants. All worker and manager names mentioned in this paper are pseudonyms; however, we have retained the actual platform names.

## 3 RESULTS

We conducted in‐depth interviews with 30 digital platform drivers who worked across nine different platforms (see Table 1) and with three digital platform managers (we attempted to recruit 10 managers but only three responded to outreach). Interviews took place between September 2020 and February 2021. This was during Wave 2 of the pandemic in Canada and before the appearance of coronavirus variants of concern.

**Table 1:** Digital platforms

*Food delivery*
*DoorDash*
*Foodora* a
*SkipTheDishes*
*UberEats*
*Package delivery*
*Amazon Flex*
*Cornershop by Uber*
*InstaCart*
*Ride‐Hail*
*Lyft*
*Uber*

^a^ Experiences before Foodora ceased Canadian operations in May 2020.

Most workers who participated (80%) were male; approximately half (52%) were members of racial minority groups; and the average age was 31 (range 21–54). Digital platform work was the primary source of employment income for 57% of workers in our sample, and the sole source for 27%. These rates are similar to those reported in a recent survey of New York City digital platform delivery drivers.^24^ On average, the drivers in our study engaged in digital platform work for 23 hours per week and earned an average of $23,790 annually from this work. Many of our participants worked across multiple apps.

Publicly available and participant‐supplied documentary data gathered included website information about the digital platform's COVID‐19‐related safety precautions and support to drivers. As well, participants provided screenshots from their smart phones that showed the digital platform's COVID‐19 advice and supports.

Below, we detail our findings regarding the five key COVID‐19 related risks faced by the digital platform drivers and how they were navigated. We first describe these worker's constant encounters with customers in the course of their work, including delivering to people in quarantine and isolation. Next, we consider safety measures, including contactless delivery, taken by the platform companies to reduce worker's exposure and ways that these had limited effectiveness. Third, we describe workers' challenge of managing customers refusing to wear masks. The fourth risk addresses time‐related pressures built into platform's algorithms and enforced by the platform's rating systems, which workers described as prompting them to take health risks. Finally, we describe the loss of income risk to workers of self‐reporting COVID‐19 symptoms to their platforms. Table 2 lays out the risks by platform type.

**Table 2:** COVID‐19 exposure and job risks

	Platform type		
	Food delivery	Package delivery	Ride hail
Close proximity with passengers for extended duration			X
Unknown health status of passenger/customer	X	X	X
Bogus passenger health screening			X
Contactless delivery not always implementable	X		
Protective equipment not always available	X	x	X
Mask‐less interactions with passenger/customers	X	X	X
Platform peer‐rating system pressures	X	X^a^	X
Platform time‐related pressures encourage safety shortcuts	X	X	
Inadequate government or platform COVID benefit pay	X	X	X
Cannot afford the wait for COVID benefits	X	X	X

^a^ Except Amazon Flex.

### 3.1 Constant customer encounters

All of the drivers in this study saw their jobs as entailing increased COVID‐19 exposure because they were required to continually interact with members of the public, some of whom could be carrying the virus. At a time when public health messaging was asking people to physically distance by staying within their “social bubbles,” ride‐hail drivers risked relatively high exposure as they were in close quarters with dozens of strangers in their car every day:

You're always meeting strangers, new people coming in and out of your vehicle, so you're definitely more exposed that way. You're not just staying in your social bubble … I think just being in the car and having random people in and out is a risk by itself (Daniella, Uber)

The food and package drivers in this study were aware that they had exposure to people with COVID‐19 as those under isolation could not leave their homes and required home deliveries. A concern for drivers across all platforms was that they lacked information about the health status of their customers. For instance, one ride‐hail driver noted that he had no information about what precautions his customers had taken or what environments they had been exposed to before entering his car:

I am transporting people, every day, that could possibly have COVID, right? So, I am always in contact with people who I have no idea what precautions they have taken in the past to protect themselves. So … every ride hail is a possibility of contracting COVID (Phil, Uber)

Driver awareness of potential exposure to the virus created health‐related anxiety about engaging in their work:

This was a customer, he opened the door, like, slightly. He looked … very, very tired, so, and runny nose and stuff … So, I was feeling very … anxious for, like, an hour after that. …. You know, like, when you feel worried … you feel that maybe … I caught the virus (Wahid, UberEats)

Based on how many people per thousand people in the population have it and how many deliveries we do, it's … highly unlikely that you could do this study for six months and not deliver to somebody that has [COVID‐19] … It's just people don't–they're not gonna say that they have it. Nobody would deliver to them, probably, if they said that (James, UberEats, SkipTheDishes)

In all, the platform drivers knew that they were potentially delivering to, or providing rides to, some people with the virus, and ride hail drivers felt especially exposed as they had customers in their cars. As they had only their own observations about which customers were ill, they were on heightened alert at all times.

### 3.2 Weak platform safety measures

The digital platform companies did make some changes to their procedures to respond to risks of platform driver exposure to COVID‐19. In particular, some ride‐hail platforms required passengers (as well as drivers) to answer health‐related screening questions. However, participants described ways that these measures had limited effectiveness. A ride‐hail manager explained that, although a platform might require passengers to provide a statement regarding absence of COVID‐19 symptoms, the health statement can be out of date by the time the passenger actually takes a ride:

I think [ride hail drivers are] more vulnerable because they're dealing with anyone and everyone. …. It's not really a place where your temperature is getting checked for every person entering the vehicle. Or there's no question of, like, ‘Do you have a cough?’ It's all through the app that's automated. But you know … these kind of verification questions of their health, in the app … are not always … current because it's not asked everyday … So that's concerning because the driver can catch [COVID‐19] very easily (Sonali, manager, Lyft)

The following quote demonstrates the inadequacy of the ride hail platform's health questionnaires for passengers:

There was this one incident where I did have a passenger who was coughing, and, like … this person had the flu. So, I really felt unsafe … but … I noticed this midway during the trip, so it was too late at that point. After that, I aired out the car, and I just went home right away (Sergio, Uber)

In this case, a driver found himself “stuck” with an ill passenger when he realized mid‐way through a ride that his passenger was symptomatic.

Contactless delivery was another measure implemented by several food delivery platform companies to reduce COVID‐19 exposures between customers and drivers. For instance, SkipTheDishes Canada instituted a policy where all deliveries, except those with alcohol, were automatically contactless, meaning that the food or package could be left at the door rather than handed directly to the customer. However, such policies did not always apply in practice to the delivery situation. With most food delivery platform companies, such as Uber Eats, it was up to the customers, not the worker, to choose the “contactless” option, leaving drivers at the mercy of the customer's choice. Even when contactless delivery was mandated, the actual encounter sometimes required contact. If an alcohol purchase was involved, then the food delivery driver had to get close to the customer to get their identification, take a picture of it, and then get the customer to sign. Contactless food delivery was also affected by pressure on workers to please the customer as a result of the tipping and peer rating systems. In the case described below, the driver stayed with a mask‐less customer while they checked their purchase because they were concerned about receiving a bad review if departing immediately after the delivery:

The person will come out without a mask and, you know, they're checking to see if everything's [all of the food items] there. … I mean, I don't want to get a bad review, so I do wait and they check that everything's there. But, you know, that also defeats the purpose of saying, like, no‐ contact delivery (Salma, Instacart)

Contactless delivery policies also made little difference to drivers who, in the course of their work, were in close contact with many different people all the way from pick‐up to drop off. For instance, food drivers described picking up food in crowded restaurants and then often having to use crowded elevators in apartment buildings to reach the drop off location. Within this process that involved multiple encounters with others in small spaces against public health advice, the final contact with the customer was a negligible part of a larger contact event:

When we are … delivering the food, a lot of the time it's at condo buildings, and then it can be hard to socially distance in those buildings, sometimes … there would be a lot of people waiting for an elevator. Sometimes there's not enough room in the elevator to keep the distance. … It's impossible to avoid being exposed (James, UberEats, SkipTheDishes)

Finally, all of the platform companies in this study provided their drivers with protective elements, such as hand sanitizer and masks. For instance, DoorDash mailed free supplies to worker's homes, if requested by them. Drivers described occasional “promotions” by Uber Eats when workers could request free protective equipment. Outside of the promotion periods, Uber Eats also provided these materials for free pick‐up; however, the platform workers described this material as located in remote warehouses that were difficult to access and not worth the time and gas expenditure. The Amazon warehouse also provided a mask and hand sanitizer to drivers when they arrived at the depot. However, the hand sanitizer portion was miniscule, for one use only. Under these conditions, drivers described opting to buy their own supplies.

### 3.3 Mask‐less customers

The ride‐hail drivers felt particularly vulnerable to COVID‐19 simply by going about the course of their work, as they had customers in their vehicle with them. However, many weighed the potential cost of this disease exposure against what they saw as the greater and more certain cost of loss of income if they did not engage in this study. One area where they regularly weighed risks was in relation to customers who refused or neglected to wear masks, which were required by digital platforms and also Ontario public health regulations. Some ride‐hail drivers in our study described feeling able to provide a “friendly reminder” to customers to wear their masks and decline passengers who refused. However, others described tolerating the risk to avoid conflict with the customer:

Even if the app clearly tells you you're required to wear a mask … some people are just stubborn … When I notice someone is not wearing a mask, it's basically a factor that's uncontrollable. You can't really tell people what to do …. At the end of the day, I was protecting myself [by not requiring masking] because you never really know what a passenger is capable of. Some of them are more aggressive (Sergio, Uber)

A key feature of most of the digital platform work (except Amazon Flex) was the workers' overall ratings, which need to be maintained above a certain threshold to stay on the app. As customer ratings of the drivers were an important part of those overall rating scores, the drivers were motivated to behave in ways that pleased the customers, including tolerating risky customer behavior. For instance, one food delivery driver noted that, while she felt unsafe when customers without face masks got very close to talking to her, she was too afraid to ask them to wear a mask or move back because they might find it rude and take back their tips.

The structure of the platforms, with peer‐ratings systems that invited customers to rate the drivers, was described by workers as creating an unequal environment that favored passengers. When weighing the cost of confronting a customer with the benefit of the customer wearing a mask, some platform workers felt that the costs outweighed the benefits.

### 3.4 Time‐related digital platform pressures

Time‐related platform pressures were another COVID‐19 exposure concern for food and package delivery drivers. That is, they had limited timeframes to deliver their goods. Time limits built into the app's algorithm put pressure on them to take safety shortcuts, such as ignoring physical distancing guidelines mandated by Ontario public health authorities to complete their tasks on time. In the following example, the driver picking up a food order in a busy restaurant weighed possible COVID‐19 exposure against the more certain risk of receiving no tip due to delayed delivery.

[T]he classic situation is you show up to a restaurant and the restaurant's very behind, and there's like a bunch of Uber drivers all waiting in the restaurant for their orders, like, crowded around each other…. There's also customers in there waiting to get their food…. Oftentimes … you have to go in and just … get your thing because it's so busy and the restaurant's so backed up. If you don't do that, you'll end up just waiting for so long, and, like the customer will be unhappy and then you won't get a tip. So yeah, there's definitely times where you kinda are … forced to go through an enclosed space where people aren't social distancing (Leyton, UberEats)

The food and shopping delivery workers seemed to always be in a rush to deliver, mainly because the timelines set by the platform's algorithm appeared to fail to account for usual delays in the course of shopping and delivery, such as road traffic and long checkout line‐ups. As well, during the pandemic the apps did not appear to add time to allow for the physical distancing that was needed, such as waiting for store aisle space to open up before reaching for food:

The app basically tells me how long it should take and what time the delivery should be delivered by. So … even without COVID, it was almost impossible to always hit that because… they don't really factor in traffic… Also…when you're in the store, it's like…if you can't find one item out of twenty … And you might have a really long line at the checkout … So, you're kind of pressured to make that up by being really fast while you're in the grocery store. I would say that's been something that I've had to stop … and just not care that I'm late with the orders …. you know, squeezing around them [other shoppers] or … being so close to them. So, if somebody's, you know, sitting there in the yogurt aisle, you kinda have to wait ‘til they move until you can go in there. … I personally just ‘take the late', but I can see where someone else might just might, to stay on time all the time, they would be exposing themselves more (Michael, Instacart)

One shopping delivery worker described feeling so uncomfortable about possible COVID‐19 exposure in a grocery store that he contacted the platform company to cancel a customer order. Despite this worker's COVID‐health‐related rationale for the order cancellation, the platform company did not give him a break on his cancellation rate, and he was penalized for canceling the order:

The person [other shopper] just wouldn't stop coughing, like, non‐stop coughing. And they had a runny nose. … Later I did find out that …they had to shut the store down for two days [because of COVID‐19]. I contacted [Instacart] support and tell them, “This is what happened…. halfway through I quit shopping because this is what I felt… I didn't feel comfortable shopping there.” And first they said, “Oh, well you have to complete the batch.” But then I explained to them again. Like, about the second time, they kind of got the vibe that I am not going to do it, so they canceled it…. When I canceled that order, that order was only … 20 bucks, so I lost that whole money. And it did get added on my cancellation rate. But my cancellation rate was only 4%, so it jumped up to 5 … You're okay up to 15 (Raju, Instacart)

In all, the time pressure built into the apps did not allow food and package delivery drivers with the time needed to reduce COVID‐19 exposure in the context of the pandemic. While some drivers accepted the consequence of loss of tips or low platform scores leading to possible platform deactivation, others accepted the COVID‐19 risk to avoid platform penalties and earn their income.

### 3.5 Risk of declaring COVID‐19 symptoms

As self‐employed workers, the drivers had to make their own decisions about what level of COVID‐19 risk to accept and bear the consequences of lost income. As a ride‐hail manager said, “It's really up to them [workers] how they control that risk” (Sonali, manager, Lyft). However, despite not being legally “employers,” the platform companies did offer to provide some financial relief to their workers as COVID‐19 increasingly became an occupational health and public health concern. For instance, Uber noted, “Any driver or the delivery person who is diagnosed with COVID‑19 or is individually asked to self‐isolate by a public health authority will receive financial assistance for up to 14 days while their account is on hold.”^39^ One ride‐hail manager, describing the platform company's provision of funds to prevent workers from working while ill, was deliberate with her words, carefully communicating this was not a sick leave policy:

We will pay them out a certain amount of money… We will compensate that driver … to incentivize them not to be on the road during…. I wouldn't call it a sick leave policy, but it's more of a benefit to encourage the driver to stay at home and take care of their wellbeing, so they're not coming out on the road (Trina, manager, Lyft)

In addition to various forms of temporary COVID‐19 sick pay for workers announced by platform companies, the Canadian government also temporarily provided emergency income support for which self‐employed workers were eligible. The Canadian Emergency Response Benefit provided up to $500 per week for workers who stopped working for reasons related to COVID‐19 and who had earned at least $5000 in the prior 12 months.^40^ However, we found that most workers in our study did not take up either of these opportunities. The compensation offered by the platforms was less than what workers would typically earn, framed accordingly by one worker as “more so incentives to keep working” (Salma, Instacart). Additionally, the workers were unsure about their eligibility for Canadian government support. For instance, some perceived that these benefits could not be accessed by self‐employed contractors if work was still available.

When asked about platform company sickness benefits, workers described being uncertain about how to apply for them or how much support they provided. They also described wanting to avoid being locked out of the platform for 14 days, which would be the case if they informed the platform company of COVID‐19 symptoms. Indeed, Cornershop and Lyft platform company managers described disconnecting workers reporting COVID‐19 symptoms for 2 weeks or deactivating their accounts until they completed isolation and could show a negative test result. Similarly, an Amazon package delivery worker described losing shifts if he mentioned that he might have been exposed to the virus.

For most digital platform companies in this study, once COVID‐19 symptoms were reported, to get back on to the app, the workers had to show proof of a negative test. While cut off the app, workers could earn no income. This was regardless of whether they ultimately tested positive for COVID‐19. At this time in Ontario, rapid tests did not exist and COVID‐19 testing was inaccessible or unavailable to certain groups or involved long lines and waiting times for results. In this context, many of these low‐wage workers saw it as an unaffordable “luxury” to take time off to test, wait days for results, and isolate, especially if a positive COVID test did not result. Isolating for 2 weeks meant no money for groceries or rent:

According to, like, public safety guidelines, you're supposed to … stop work and self‐isolate and get tested and all that. But that is up to the individual. And while I probably would … a lot of the people that do this kind of work do not have that luxury. If they don't take that shift, they don't eat next week. So, they work (Arjun, Amazon Flex, DoorDash)

So, whenever I used to feel sick, I used to take a day or two off just to try to recover, and then I go back to work directly. But I really can't stop working. I know, if you're sick you have to stay home and don't work. But my landlord wouldn't understand this, and she want to get to her rent at the end of the month (Derrick, Instacart, Skip, Doordash)

Other workers self‐managed their illness rather than staying home as per platform and public health recommendations. This driver chose to hide her cough to not lose her shifts:

Basically, if you can try to avoid the coughing, it's okay …. You don't cough in the warehouse. … If you cough, and someone will suspect a problem, so you may lose your shift…. Usually, if I try to cough, I only try to go to washroom or the car, nothing else (Sammy, Amazon Flex)

Rather than inform their platform company of their health symptoms and risk being deactivated from the digital platform, workers made personal decisions about when to stay off work and how soon to return:

Actually, I had large fever in August … just one day, and I recovered. But I don't think it's COVID 19, so… Nothing related to COVID… That day I was off… Next day I recovered (Charles, Doordash)

I've had the sniffles for a couple of days, but I don't think it was Corona. I haven't been tested, though, so, who knows…I think I probably took those days off just because I wasn't feeling well… They [the apps] do say that if you are exhibiting any symptoms–and they give you the list of symptoms–that you don't work … But especially… when it's colder weather…you're gonna get the sniffles when you're out biking… So you can't just take every day off (Brad, UberEats, Foodora)

In all, despite platform companies and the Canadian government offering financial compensation for time off due to COVID‐19, drivers largely did not take this up. Barriers to accessing this support largely centered on uncertainty about the application process, insufficient funds provided by the benefit, and workers' inability to wait without income for a benefit cheque to arrive.

## 4 DISCUSSION

The aim of our study was to identify perceived COVID‐19‐related exposure and job risks faced by digital platform drivers and to document the measures in place to protect the health of these workers. By engaging in in‐depth interviews with platform workers and managers, we were able to gain a grounded understanding of the COVID‐19‐related conditions of driver's work as well as their motivations and rationales for their actions related to COVID‐19 risk‐taking and protection. This study extends the limited literature on platform work and COVID‐19 risks faced by digital platform drivers.^35^ We also provide recommendations on occupational health interventions for this sector.

### 4.1 Interventions to prevent COVID‐19 occupational exposure and job risk

Our in‐depth interviews with Canadian digital platform drivers and managers led to the identification of five key COVID‐19‐related exposure and job risks that drivers faced in the course of their work. Key exposure risk was the nature of ride‐hail, package, and food delivery work, as it involved frequent contact with customers, some of whom would inevitably be virus carriers. Although the digital platform companies instituted COVID‐19 risk‐reduction measures for drivers, such as customer health statements, contactless delivery, and provision of masks and hand sanitizers, drivers and managers described many ways that these measures were ineffective or difficult to implement. Given airborne transmission of the virus and public health mask mandates, mask‐less customers also posed an exposure risk to drivers, but many felt that it was not worth refusing or contesting these customers because of the possibility of losing tips or getting a bad rating from the customer. Time‐related platform pressures were a further risk encountered by the drivers. The need to rush to get packages and groceries delivered on time prompted drivers to breach public health guidelines requiring 2‐metre physical distancing. Finally, we found that, despite digital platform companies and the Canadian government providing income support to COVID‐19‐positive workers, some drivers did not take this up because they could not afford the wait for benefits or risk the loss of income while denied access to the platform should they self‐report and be deactivated.

There is emerging literature focused on health experiences and risks to digital platform workers during the COVID‐19 pandemic, which echoes our findings. Blundell et al.^1^ found that 78% of United Kingdom platform workers felt their health to be at risk while working but they continued to work due to economic insecurity. Similarly, Beckman et al.^35^ found that Seattle, USA, drivers were very concerned about COVID‐19 exposure, and experiencing high stress. Only one‐third of the workers surveyed had been provided with free masks or hand sanitizer. An Uber survey of their American drivers found strong concerns among drivers about their physical and mental health during the COVID‐19 pandemic.^41^ The stress of COVID was widespread: in an international survey of digital platforms, Howson et al.^19^ note the mental stress placed on workers due to the asymmetrical power of platforms and the lack of medical treatment available for COVID‐infected workers in many jurisdictions.

We found that, as self‐employed workers, platform drivers were generally expected to educate themselves about occupational risk and to avoid dangerous situations (“It's really up to them”). Such downloading of risks to workers is a key characteristic of precarious employment conditions.^10^ The part‐time and limited‐term aspects of precarious employment also make it difficult for workers to form relationships to protect their health by collective action. Despite these barriers, platform workers have, in some cases, successfully unionized.^42^, ^43^

In the following parts of this discussion, we identify ways that digital platform companies and governments could make changes to address the risks identified in this study.

### 4.2 Platform‐level interventions

A key intervention point for digital platform companies is loosening *time pressures on workers* and waiving cancellation penalties related to driver's perceived COVID‐19 risk. Each of these measures would provide workers with more latitude to make decisions to avoid COVID‐19 exposure risk. Time leeway to permit proper physical distancing seemed noticeably absent in the platform company's algorithms for the workers in our study, leaving them rushed and risking COVID‐19 exposure in crowded areas to meet timeline requirements. Waived cancellation penalties would give space to drivers to deny service to *mask‐less customers*. It is possible to engineer algorithms to account for occupational risks. As argued by Todolo‐Signes,^44^ algorithms can and should be programmed to take into account all known occupational health risks. The content and transparency of digital platform algorithms is an issue taken up by the European Commission's proposed directive on improving the working conditions of people working through digital labor platforms. The directive, “increases transparency in the use of algorithms by digital labor platforms, ensures human monitoring on their respect of working conditions and gives the right to contest automated decisions.”^7^,p.1.

*Platform safety measures* could also be improved. Ride‐hail companies' screening of customers could be time‐sensitive; for instance, required on the same day as the ride. When public health requires physical distancing, all platform companies should require that customers use contactless delivery, rather than leaving the choice to the customer's discretion. As well, contactless delivery could be extended to alcohol deliveries by creating remote electronic scanning of customer identification. It is surprising, given the technological sophistication of platform companies, that this innovation was not already in place. Finally, in a context where our study and others^19^, ^35^ have found that personal protective equipment was not always easily available, platform companies could provide more easily accessible personal protective equipment to workers, while also ensuring that it is of high quality.

Providing sick pay that is accessible and not financially punitive (e.g., being locked out of the platform pre‐diagnosis) is another way that platform companies could better support the health of their workers. Ensuring that workers have an income while ill would reduce *the workers' risk of declaring COVID‐19 symptoms*. Although platforms did set up temporary COVID‐19 sick pay for workers, these financial interventions did not appear to prevent many platform workers in our study from working while ill with COVID‐19, and consequently, possibly exposing the public to the virus.

Although the improvements to platform conditions detailed above might improve working conditions for drivers, there is currently little legal pressure on platform companies to make such changes. With significant lobbying activity,^45^, ^46^ platform companies have been largely successful at avoiding employer status and responsibilities that come with it, including employment and occupational health standards.

### 4.3 Government‐level interventions

Government‐level legal and policy interventions are especially important for improving the occupational health and safety of digital platform workers, as these interventions have the weight of enforcement by public authorities. However, an important context for platform work is neoliberal approaches to government policy that, since the 1980s, have favored free market capitalism and deregulation.^47^ Over this time, advanced economies have seen a broad erosion of employment and income security and a growth of low wage, part‐time, and insecure employment.^10^ Given limited government protections, the financial insecurity of platform drivers in our study prompted them to take health risks that, in turn, increased public exposure to the virus (e.g., working while ill).

To their credit, the Canadian government introduced the temporary Canadian Emergency Relief Benefit that, unlike past social security supports, included all self‐employed among those eligible to claim benefits. However, workers in our study mostly did not take this up. Indeed, during the pandemic, advocates for low wage workers emphasized that programs providing delayed benefits are ineffective because workers cannot afford to wait to apply for and receive benefits; instead, they need paid sick days.^48^ With paid sick days, workers would be able to take time off when ill because income support would flow continuously. Our findings are similar to those of platform workers in the US, who were found to not use available employment support benefits under the CARE Act because of long benefit wait times or lacking documentation.^19^, ^49^ Ravenelle et al.'s^49^ survey of gig workers in New York City further found that platform workers often did not know they were eligible for support. They assumed that working part‐time, being on a temporary contract, or being an immigrant (noncitizen) rendered them ineligible. Interventions to address the problem of workers not declaring COVID‐19 symptoms because of the job‐and income‐related *risk of declaring COVID‐19 symptoms* would need to focus on the basic issues of income security and labor standards for low‐wage workers.^50^ For instance, governments might introduce a basic income program, which might provide workers with sufficient leeway to resist poor working conditions.^51^

Following the recently proposed European Commission directive on improving working conditions of platform work, we suggest that platform drivers should have full employee status, with all of its associated rights and protections, when the work they do is not truly independent of control by the digital platform.^7^ The “employer” status of Foodora was established in an Ontario ruling on the right to be unionized, which also determined that driver's relationships with Foodora most closely resembled an employment relationship. Foodora withdrew from Canada immediately after this ruling.^52^ Similar legal battles have ensued in the USA, including a law passed in 2019 in California requiring platform companies like Uber to hire their drivers, which was then ruled unconstitutional in 2021.^53^

Recognition of platform drivers as “employees” rather than self‐employed would be an upstream preventive strategy that would make a positive contribution to government tax pools that, in turn, support all workers. That is, as employers, digital platform companies would be required to contribute to social security programs like employment insurance and pension plans.^22^ Consequently, as employees, when platform workers become injured or ill, they would be able to access workers' compensation instead of needing to resort to basic social assistance payments.^19^, ^54^

Measures to protect the health of platform workers would have carry‐on effects on public health. That is, COVID‐19 exposure risk for workers translates into broader public health risks if drivers became infected with the virus and then pass it on to customers, families, and consequently, the wider population. As such, government measures to better protect the health of platform workers would also constitute important public health measures.

### 4.4 Future work and conclusion

We recommend that future research on the occupational health of digital platform workers measure the extent of worker presenteeism, especially in relation to the transmission of contagious viruses. The effectiveness of occupational health interventions, such as income support benefits when ill, could also be measured among platform workers. What makes platform workers different from regular workers are pervasive and rather impersonal algorithmic digital platform pressures that nudge them toward accepting health risks, which creates a need to examine the effectiveness of occupational health interventions among this specific population. Future work could also examine the crossover effects of COVID‐19 occupational health protections on public health outcomes. While platform work has been found to be gendered and racialized,^55^, ^56^ our study did not discern specific gender or race effects in relation to COVID‐19 risks faced by drivers. It may be that low income created a common thread among participants, or that our qualitative study did not access a sufficient sample to explore these dimensions.

Strengths of our study include the qualitative approach that enabled us to identify a range of perceived COVID‐19‐related risks among digital platform workers and why the current strategies to mitigate risk appeared to be largely ineffective. Our findings provide information that can be used to develop future larger‐scale studies regarding risk and to improve prevention strategies. Our study does not aim for statistical generalization; rather, our goal was to provide enough detail for analytic generalization, in which the reader is provided with sufficient information to decide whether the findings are relevant to other contexts or jurisdictions.^57^ It is important to note that interventions to address perceived risks may not comprehensively address all relevant risks, including risks not directly perceived by workers.

In all, our study found that the nature of digital platform driving work was one of constant possible exposure to COVID‐19 through work processes that created contact with customers and regular encounters with crowded environments. Despite digital platform company attempts to screen passenger health, institute contactless delivery, and provide financial compensation to workers when ill with COVID‐19, we found that these had limited impact on the day‐to‐day COVID‐19 exposure and job risks faced by digital platform drivers. It is important to consider that, as self‐employed workers without the protection of employment and occupational health standards, platform workers are absorbing most of the occupational risks related to COVID‐19. This arrangement also poses a societal risk because platform workers who take risks because of digital platform pressures can also spread the virus in the course of their work delivering food, packages, and people.

## AUTHOR CONTRIBUTIONS

EllenMacEachen, Samantha B.Meyer, and ShannonMajowicz conceived of and designed the funded research proposal that guided and supported this study. Pamela Hopwood, Meghan Crouch, Joyceline Amoako, Yamin T. Jahangir, and Antonela Ilic gathered interview and documentary data. All authors contributed to the data analysis. EllenMacEachen drafted this manuscript with feedback from all authors. All authors approve this submitted version and are accountable for this study.

## CONFLICT OF INTEREST

The authors declare that there are no conflict of interest.

## DISCLOSURE BY AJIM EDITOR OF RECORD

John Meyer declares that he has no conflict of interest in the review and publication decision regarding this article.

## ETHICS APPROVAL AND INFORMED CONSENT

Ethics approval for this study was provided by the University of Waterloo Research Ethics Board (REB #42448). Following the University of Waterloo COVID‐19 research requirements, informed consent was provided verbally.

## Data Availability

The data that support the findings of this study are available on request from the corresponding author. The data are not publicly available due to privacy or ethical restrictions.

## References

[1] Blundell J , Machin S , Ventura M . Covid‐19 and the self‐employed: six months into the crisis. *COVID‐19 Analysis Series*. 2020. Accessed January 12, 2022. https://cep.lse.ac.uk/pubs/download/cepcovid-19-012.pdf

[2] Greenhalgh T , Jimenez JL , Prather KA , Tufekci Z , Fisman D , Schooley R . Ten scientific reasons in support of airborne transmission of SARS‐CoV‐2. Lancet. 2021;397(10285):1603‐1605. 33865497 10.1016/S0140-6736(21)00869-2 PMC8049599

[3] Mead GH . Moments of Thought in the Nineteenth Century. University of Chicago Press; 1936.

[4] Mastercard, Kaiser Associates . The global gig economy: capitalizing on a ~$500B opportunity. *Mastercard gig economy industry outlook and needs assessment*. 2019. Accessed January 11, 2021. https://newsroom.mastercard.com/wp-content/uploads/2019/05/Gig-Economy-White-Paper-May-2019.pdf

[5] Aston J , Vipond O , Virgin K , Youssouf O . Retail e‐commerce and COVID‐19: how online shopping opened doors while many were closing. *StatCan COVID‐19: data insights for a better Canada*. Catalogue no. 45280001. pp. 1‐7; 2020.

[6] ECommerce Europe . *European E‐commerce report*. p. 111; 2021. Accessed January 20, 2022. https://ecommerce-europe.eu/wp-content/uploads/2021/09/2021-European-E-commerce-Report-LIGHT-VERSION.pdf

[7] European Commission . Commission proposals to improve the working conditions of people working through digital labour platforms. *Improving working conditions in platform work*. Press release. December 9, 2021. Accessed December 20, 2021. https://ec.europa.eu/commission/presscorner/detail/en/ip_21_6605

[8] Jeon S‐H , Liu H , Ostrevsky Y . Measuring the gig economy in Canada using administrative data. *Analytical Studies Branch*. December 16, 2019. Accessed Aprail 26, 2022. https://www150.statcan.gc.ca/n1/en/pub/11f0019m/11f0019m2019025-eng.pdf?st=qtsv4V

[9] Lewchuk W . Precarious jobs: where are they, and how do they affect well‐being? Econ Labour Relat Rev. 2017;28(3):402‐419.

[10] Kalleberg AL . Precarious work, insecure workers: employment relations in transition. Am Sociol Rev. 2009;74(1):1‐22. 10.1177/000312240907400101

[11] Kreshpaj B , Orellana C , Burström B , et al. What is precarious employment? A systematic review of definitions and operationalizations from quantitative and qualitative studies. Scand J Work, Environ Health. 2020;46(3):235‐247. 31901944 10.5271/sjweh.3875

[12] Bhattacharya A , Ray T . Precarious work, job stress, and health‐related quality of life. Am J Ind Med. 2021;64(4):310‐319. 33543533 10.1002/ajim.23223 PMC9904539

[13] Benavides FG , Silva‐Penaherrera VA . Informal employment, precariousness, and decent work: from research to preventive action. Scand J Work Environ Health. 2022;48(3):169‐172. 35289374 10.5271/sjweh.4024 PMC9523463

[14] Berg J , Furrer M , Harmon E , Rani U , Silberman MS . *Digital labour platforms and the future of work: towards decent work in the online world* (Report). International Labour Organization; 2018. Accessed January 4, 2022. https://www.ilo.org/wcmsp5/groups/public/---dgreports/---dcomm/--publ/documents/publication/wcms_645337.pdf

[15] Veen A , Barratt T , Goods C . Platform‐capital's “app‐etite” for control: a labour process analysis of food‐delivery work in Australia. Work, Employ Soc. 2020;34(3):388‐406.

[16] De Stefano V , Durri I , Stylogiannis C , Wouters M . *Platform work and the employment relationship*. International Labour Organization; 2021. Working paper 27. Accessed Febuary 2, 2022. https://www.ilo.org/wcmsp5/groups/public/---ed_protect/---protrav/---travail/documents/publication/wcms_777866.pdf

[17] Bartel E , MacEachen E , Reid‐Musson E , et al. Stressful by design: exploring health risks of ride‐share work. J Transp Health. 2019;14:1‐12.

[18] Warren T . Work–life balance and gig work: “where are we now” and “where to next” with the work–life balance agenda? J Ind Relat. 2021;63(4):522‐545.

[19] Howson K , Spilda FU , Bertolini A , et al. Stripping back the mask: working conditions on digital labour platforms during the COVID‐19 pandemic. *Int Labour Rev*. 2021;1‐35. 10.1111/ilr.12222 PMC8444801 34548681

[20] Nilsen M , Kongsvik T , Almkov PG . Splintered structures and workers without a workplace: how should safety science address the fragmentation of organizations? Saf Sci. 2022;148:105644.

[21] Heeks R , Graham M , Mungai P , Van Belle JP , Woodcock J . Systematic evaluation of gig work against decent work standards: the development and application of the fairwork framework. Inf Soc. 2021;37:5‐286.

[22] Behrendt C , Nguyen QA , Rani U . Social protection systems and the future of work: ensuring social security for digital platform workers. Int Soc Secur Rev. 2019;72:3‐41.

[23] Mishel L . Uber and the labor market: Uber drivers' compensation, wages, and the scale of Uber and the gig economy. May 15, 2018. Accessed January 11, 2021. https://www.epi.org/files/pdf/145552.pdf

[24] Figueroa M , Guallpa L , Wolf A , Tsitouras G , Hernandez HC . *Essential but unprotected: App‐based food couriers in New York City*. p. 47; 2021. Accessed January 12, 2022. https://img1.wsimg.com/blobby/go/6c0bc951-f473-4720-be3e-797bd8c26b8e/09142021CHARTSLos%20Deliveristas%20Unidos-v02.pdf

[25] Woodcock J , Graham M . The Gig Economy: A Critical Introduction. Polity Press; 2019.

[26] Katta S , Badger A , Graham M . (Dis)embeddedness and(de)commodification: COVID‐19, Uber, and the unravelling logics of the gig economy. Dialogues in Human Geography. 2020;10(2):203‐207.

[27] MacEachen E , de Rijk A , Dyreborg J , et al. How do laws, policies and collective agreements protect the health and safety of low‐wage and digital platform workers? Lessons learned during the COVID‐19 pandemic. 2022. 10.1177/10482911221133796 PMC9582739 36262099

[28] U.S. Department of Treasury . About the CARES Act and the Consolidated Appropriations Act. Accessed February 4, 2022. https://home.treasury.gov/policy-issues/coronavirus/about-the-cares-act

[29] Government of Canada . Canada emergency response benefit. Updated April 24, 2020. Accessed May 10, 2020. https://www.canada.ca/en/services/benefits/ei/cerb-application.html

[30] Government of Ontario . *Backgrounder: Working for Workers Act, 2022*. Office of the Premier. 2022.

[31] Stanford J . Don't be fooled by Ontario's “minimum wage” for gig workers. February 28, 2022. Accessed April 28, 2022. https://centreforfuturework.ca/2022/02/28/dont-be-fooled-by-ontarios-minimum-wage-for-gig-workers/

[32] Moulds J . Gig workers among the hardest hit by coronavirus pandemic. Vol SDG08. *Futute of Work*. April 21, 2020. Accessed January 11, 2021. https://www.weforum.org/agenda/2020/04/gig-workers-hardest-hit-coronavirus-pandemic/

[33] Watters H . What it's like for couriers who keep delivering so you can stay home. *CBC*. Accessed January 11, 2021. https://www.cbc.ca/news/canada/toronto/couriers-delivery-covid-pandemic-1.5523482

[34] Needleman C . Qualitative methods for intervention research. Am J Ind Med. 1996;29:4‐337. 10.1002/(SICI)1097-0274(199604)29:4<329::AID-AJIM10>3.0.CO;2-3 8728134

[35] Beckman KL , Monsey LM , Archer MM , Errett NA , Bostrom A , Baker MG . Health and safety risk perceptions and needs of app‐based drivers during COVID‐19. Am J Ind Med. 2021;64(11):941‐951. 34523153 10.1002/ajim.23295 PMC8653178

[36] Clarke AE , Friese C , Washburn RS . Situational Analysis: Grounded Theory After the Interpretive Turn. 2nd ed. Sage; 2018.

[37] Patton MQ . Purposeful sampling. In: Patton MQ , ed. Qualitative Evaluation and Research Methods. Sage; 1990:169‐186.

[38] Seale C . Quality in qualitative research. In: Lincoln YS , Denzin NK , eds. Turning Points in Qualitative Research: Tying Knots in a Handkerchief. AltaMira; 2003:169‐184.

[39] Uber. *Coronavirus (COVID‐19) resources & updates*. Accessed April 28, 2020. https://www.uber.com/ca/en/coronavirus/

[40] Canada Go . *Canada emergency response benefit*. 2020. Accessed January 22, 2021. https://www.canada.ca/en/services/benefits/ei/cerb-application.html

[41] Uber . *Uber Experience Survey*. 2020. Accessed January 19, 2022. https://uber.app.box.com/s/ilxsiqy0bkfhgum8o15n6k6bqi2rqn9c

[42] Tassinari A , Maccarrone V . Riders on the storm: workplace solidarity among gig economy couriers in Italy and the UK. Work, Employ Soc. 2020;34(1):35‐54.

[43] Lane M . Regulating digital platform work in the digital age. *OECD Going Digital Toolkit Policy Note*. p. 23; 2020. https://goingdigital.oecd.org/toolkitnotes/regulating-platform-work-in-the-digital-age.pdf

[44] Todolo‐Signes A . Making algorithms safe for workers: occupational risks associated with work managed by artificial intelligence. Transfer. 2021;27:433‐452.

[45] Beretta M . *How is tech lobbying shaping federal policy*? Policy Options, Institute for Research on Public Policy; 2019.

[46] Chan W . “Insidious and seductive”: Uber funds new lobbying group to deny rights for gig workers. *The Guardian*. Accessed May 2, 2022. https://www.theguardian.com/business/2022/mar/11/uber-funds-new-lobbying-group-to-deny-rights-for-gig-workers

[47] Albert M . Capitalism Against Capitalism. Whurr Publishers; 1993.

[48] *Decent Work and Health Network. Before it's too late: how to close the paid sick days gap during COVID‐19 and beyond*. p. 49; 2021. Accessed January 20, 2022. https://d3n8a8pro7vhmx.cloudfront.net/dwhn/pages/135/attachments/original/1604082294/DWHN_BeforeItsTooLate.pdf?1604082294

[49] Ravenelle AJ , Kowalski KC , Janko E , Rambotti S , Davis AP , Hill TD . The side hustle safety net: precarious workers and gig work during COVID‐19. Sociol Perspect. 2021;64:5898‐5919.

[50] Hauben H , Lenerts K , Kraatz S . *Platform economy and precarious work: Mitigating risks*. Policy Department for Economic, Scientific and Quality of Life Policies Directorate‐General for Internal Policies. p. 12; 2020. chrome‐extension://efaidnbmnnnibpcajpcglclefindmkaj. AccessedMay 1, 2022. https://www.europarl.europa.eu/RegData/etudes/BRIE/2020/652721/IPOL_BRI(2020)652721_EN.pdf

[51] Stahl C , MacEachen E . Universal basic income as a policy response to COVID‑19 and precarious employment: potential impacts on rehabilitation and return‑to‑work. J Occup Rehabil. 2021;31(1):3‐6. 32816204 10.1007/s10926-020-09923-w PMC7439237

[52] Richards MS , Radojcic A , Seal J . The rubber hits the road: the Ontario Labour Relations Board holds that Foodora couriers are ‎dependent contractors. *Can LII Connects*; 2020.

[53] Conger K , Browning K. A judge declared California's gig worker law unconstitutional. Now what? *New York Times*.

[54] MacEachen E . Digital “gig work” companies are getting a free ride on the backs of taxpayers. *Toronto Star*. Accessed November 30, 2021. https://www.thestar.com/opinion/contributors/2021/11/25/digital-gig-work-companies-are-getting-a-free-ride-on-the-backs-of-taxpayers.html

[55] van Doorn N . Platform labor: on the gendered and racialized exploitation of low‐income service work in the “on‐demand” economy. Inf, Commun Soc. 2017;20:898‐914. 10.1080/1369118X.2017.1294194

[56] Cook C , Diamond R , Hall J , List JA , Oyer P . *The Gender Earnings Gap in the Gig Economy: Evidence from over a Million Rideshare Drivers*; 2018. Working paper 3637.

[57] Ruddin LP . You can generalise, stupid! Social scientists, Bent Flyvberg, and case study methodology. Qual Enquiry. 2006;12(4):797‐812.

